# The best PEEP or the optimal PEEP or the piece PEEP of the mechanical power puzzle?

**DOI:** 10.1186/s13054-022-04162-2

**Published:** 2022-10-03

**Authors:** George Briassoulis, Panagiotis Briassoulis, Stavroula Ilia

**Affiliations:** 1 Postgraduate Program “Emergency and Intensive Care in Children Adolescents and Young Adults”, Pediatric Intensive Care Unit, School of Medicine, University of Crete, Section 6D (Delta), Office 03, Voutes, 71003 Heraklion, Crete Greece; 2 2nd Department of Anaesthesiology, School of Medicine, Attikon Hospital, National and Kapodistrian University of Athens, Athens, Greece

**Dear Editor,**

We read with great interest the brief report by Dr. Rezaiguia‐Delclaux et al. comparing driving pressure and absolute PaO_2_/FiO_2_ ratio in determining the best positive end‐expiratory pressure (PEEP) level in patients with moderate (54%) or severe (46%) acute respiratory distress syndrome (ARDS) [1]. PEEP was increased until plateau pressure reached 30 cmH_2_O at constant tidal volume and then decreased at 15‐min intervals, to 15, 10, and 5 cmH_2_O. The best PEEP by PaO_2_/FiO_2_ ratio (PEEP_O2_) was defined as the highest PaO_2_/FiO_2_ ratio obtained, and the best PEEP by driving pressure (PEEP_DP_) as the lowest driving pressure. The best mean PEEP_O2_ value was 11.9 ± 4.7 cmH_2_O compared to 10.6 ± 4.1 cmH_2_O for the best PEEP_DP_, while only 37.7% of PEEP levels were the same with the two methods. Although the best PEEP maintains the recruitment of unstable alveoli, improving oxygenation and lung compliance, the study concludes that depending on the method chosen, the best PEEP varies [1]. There are issues that we want to highlight and comment on.

First, in this non-typical cohort of ARDS patients, 60% were admitted after thromboendarterectomy and lung transplantation. It would have been interesting to know what lung mechanics changes and hemodynamic consequences were by applying the two methods in this cohort of patients. Not only the “oxygenation method” may not reliably protect against hyperinflation, but also, setting FiO_2_ at toxic levels (> 0.6) increases oxidative stress. The concept of ventilator-associated condition (VAC) is defined as a “preventable harm” which causes deterioration in pulmonary status and the need for increasing ventilatory support. Shouldn't these patients have been placed in a prone position to increase the homogeneity of ventilation and prevent VAC?

Second, if the dominant mechanism of hypoxemia is not alveolar collapse, by blindingly increasing PEEP to improve the PaO_2_/FiO_2_ ratio the energy transferred to the lung parenchyma might lead to dynamic hyperinflation maximizing ventilation-induced lung injury (VILI) and impairing cardiac output. Although optimal levels of PEEP should be set according to lung disease to prevent injury, due to cyclical opening and collapse of the alveoli (atelectatic trauma), inhomogeneous areas act as local stress multipliers doubling the stress compared to that present in other parts of the same lung. Furthermore, like COVID-19 patients, the mechanisms of hypoxemia in the thromboendarterectomy and lung transplanted patients might have been caused by pulmonary endothelial dysfunction rather than by altered pulmonary mechanics. Personalizing PEEP to target optimal compliance might identify ARDS patients with functional lung units, avoiding the indiscrete application of high PEEP [2]. Using the nitrogen washout/washin technique, we have recently demonstrated that end-expiratory lung volume (EELV), but not compliance (Crs), increased from PEEP 4 to PEEP 10 cmH_2_O (*p* = 0.001) in ARDS pediatric patients longitudinally [3]. Previous experimental and clinical studies have similarly questioned the reliability of Crs for lung recruitment/derecruitment estimation in ARDS.

Third, the study lacks a control (non-ARDS or at-risk of ARDS) group and does not show comparative results between sub-cohorts, based on the PaO_2_/FiO_2_ ratio or driving pressure. Unexpectedly, the PEEP effect was tested in the very short term. In a total of 896 measurements calculated in 32 mechanically ventilated subjects, we showed that time interacted differently with the two pediatric ARDS phenotypes at PEEP 4 and 10 cmH_2_O. In ARDS, strain and stress increased by 24 h, remaining within safe limits, and declined by 72 h at PEEP 10 (*p* = 0.02).In the at-risk group, strain and stress declined steadily from 6 to 72 h at PEEP 10 (*p* = 0.001) [4].

It has been previously demonstrated that the mechanical power is similar at 5 and 15 cmH_2_O PEEP both in normal subjects and in ARDS patients (*p* < 0.0001) and that it increases linearly with PEEP and exponentially with other ventilatory settings [5]. Looking for the most suitable combination of variables involved in avoiding “ergotrauma,” automated ventilators with smart monitoring tools measuring pulmonary physiology with implemented artificial intelligence and equipped with equations calculating mechanical power, are eagerly expected.

## Data Availability

Not applicable.
