# Supplementary material for: The best PEEP or the optimal PEEP or the piece PEEP of the mechanical power puzzle?
Source: Crit Care. 2022 Oct 3;26:298. doi: 10.1186/s13054-022-04162-2 (PMC9527713; doi:10.1186/s13054-022-04162-2)
Supplement: Related texts

# Author’s reply to: The best PEEP or the optimal PEEP or the piece PEEP of the mechanical power puzzle?

**Authors:** François Stéphan, Saida Rezaiguia-Delclaux

**Affiliations:** 3 grid.414221.0 Cardiothoracic Intensive Care Unit, Hôpital Marie Lannelongue, 133 Avenue de la Résistance, 92350 Le Plessis-Robinson, France; 4 grid.460789.4 0000 0004 4910 6535 School of Medicine, Paris-Saclay University, Kremlin-Bicêtre, France; 5 grid.414221.0 INSERM U999, Pulmonary Hypertension: Pathophysiology and Novel Therapies, Hôpital Marie Lannelongue, Le Plessis-Robinson, France

**Dear Editor,**

We thank Briassoulis et al. for their interest in our study [1] and for their pertinent remarks.

We acknowledge that our study was performed on a very specific population. However, it should be noted that filling pressures remain low in these reperfusion edemas, even though extravascular water increases on the 2nd postoperative day [6].

Unfortunately, due to the design of the study, where the best PEEP determined by driving pressure was scored following the oxygenation-based PEEP trial, we were not able to evaluate the lungs mechanics changes and hemodynamic consequences.

The authors also point out the possible impact of the level of FiO_2_ adjusted after the PEEP trial. This median FiO_2_ level was equal to 0.70 [IQR 0.6–0.8] in our study. The adjustment of FiO_2_ is still a matter of controversy [7, 8]. The lung of patients with ARDS might exhibit some relative resistance to prolonged oxygen exposure [7]. Last, after the PEEP trial, 29 patients (23.8%) were placed in the prone position.

We agree on the mechanisms of hypoxemia in our population. As suggested by the authors, it would be interesting to study the pulmonary mechanics in our population and in other populations of patients with ARDS.

Finally, the authors point out the absence of a group of patients without ARDS. In clinical practice, we do not perform PEEP trials in patients without ARDS. We agree with the authors that the level of PEEP is a dynamic process and that the level of PEEP may vary with time. This raises the question of performing a new PEEP trial 24–48 h later.

The setting of PEEP will still give rise to many debates among the intensivists. Although no one method has been clearly shown to be superior to another, it seems logical that this level of PEEP should be based on an objective rather than a random method.
